# Identification, modification, and implementation of an evidence-based psychotherapy for children in a low-income country: the use of TF-CBT in Zambia

**DOI:** 10.1186/1752-4458-7-24

**Published:** 2013-10-23

**Authors:** Laura K Murray, Shannon Dorsey, Stephanie Skavenski, Margaret Kasoma, Mwiya Imasiku, Paul Bolton, Judith Bass, Judith A Cohen

**Affiliations:** 1 Department of Mental Health, Bloomberg School of Public Health (JHSPH), Applied Mental Health Research Group, Johns Hopkins University, 624 N. Broadway Street, 8th Floor, Baltimore, MD 21205, USA; 2 University of Washington, Department of Psychology, 335 Guthrie Hall, Seattle, WA 98195, USA; 3 SHARPZ Organization, Lusaka, Zambia; 4 Department of Psychology, University Teaching Hospital, Lusaka, Zambia; 5 JHSPH, Applied Mental Health Research Group, Department of International Health, 615 N. Wolfe Street, 8th Floor, Baltimore, MD 21205, USA; 6 Temple University School of Medicine, Allegheny General Hospital, 4 Allegheny Center, 8th Floor, Pittsburgh, PA 15212, USA

**Keywords:** Child trauma, Evidence-based treatment, Implementation, International, Low-resource setting

## Abstract

**Background:**

The need to address the treatment gap in mental health services in low- and middle-income countries (LMIC) is well recognized and particularly neglected among children and adolescents. Recent literature with adult populations suggests that evidence-based mental health treatments are effective, feasible, and cross-culturally modifiable for use in LMIC. This paper addresses a gap in the literature documenting pre-trial processes. We describe the process of selecting an intervention to meet the needs of a particular population and the process of cross-cultural adaptation.

**Methods:**

Community-based participatory research principles were implemented for intervention selection, including joint meetings with stakeholders, review of qualitative research, and review of the literature. Trauma-focused Cognitive Behavioral Therapy (TF-CBT) was chosen as the evidence-based practice for modification and feasibility testing. The TF-CBT adaptation process, rooted within an apprenticeship model of training and supervision, is presented. Clinical case notes were reviewed to document modifications.

**Results:**

Choosing an intervention can work as a collaborative process with community involvement. Results also show that modifications were focused primarily on implementation techniques rather than changes in TF-CBT core elements.

**Conclusions:**

Studies documenting implementation processes are critical to understanding why intervention choices are made and how the adaptations are generated in global mental health. More articles are needed on *how to* implement evidence-based treatments in LMIC.

## Background

Mental health is a priority global health issue [1,2]. The most recent Disability-Adjusted Life Years (DALYs; a metric used to quantify the burden of diseases, injuries, and risk factors) report continues to show the high burden of mental health disorders [3]. Despite this, a well-recognized and substantial mental health need and treatment gap exists in low- and middle-income countries (LMIC). In some LMIC, this gap exceeds 90% [4,5]. The gap is most severe for children and adolescents [6]. In attempts to reduce this gap, there is an increasing amount of research examining the effectiveness of psychological treatments for different populations and settings. A few researchers have specifically examined the effectiveness in LMIC of evidence-based treatments (EBTs) developed in high-income countries. Randomized clinical trials (RCTs) have demonstrated positive outcomes for common mental health problems (i.e., depression, anxiety, and PTSD) and functioning for a range of EBTs. Examples include: 1) Interpersonal Psychotherapy (IPT) conducted in Uganda [7,8] and India [9]; 2) Cognitive Behavioral Therapy (CBT) in Pakistan [10]; 3) Cognitive Processing Therapy (CPT) in Democratic Republic of Congo [11] and Iraq Manuscripts in submission and 4) Common Elements Treatment Approach (CETA) in Iraq [13] and Thailand Cognitive Processing Therapy. Notably, all but one study [8] was with adult populations, further highlighting the dearth of literature addressing child mental health problems.

All of these studies employed a task-shifting/sharing model, in which lay counselors, with little to no mental health experience, delivered the EBT. As the body of research on EBT in LMIC continues to grow, there has been increased attention and interest in the potential of task-shifting EBT to address the mental health treatment gap. For example, the World Health Organization (WHO) recommends EBTs as frontline treatments for moderate to severe symptoms of multiple mental health disorders in its Mental Health Gap Action Programme (mhGAP) [12].

The use of EBTs in LMIC has been both heralded, given positive outcomes in RCTs, and controversial, as treatments are typically developed and transported from high-resource, “western” countries or contexts, which are culturally and contextually distinct. In the literature, some are of the opinion that efforts to address the treatment gap in LMIC should build and benefit from evidence in other settings (e.g. high-resource countries) [12,13]. Equally present is the viewpoint that efforts should take a more ethnographic approach and identify and test local strategies of addressing mental health problems [14,15]. Addressing the substantial mental health treatment gap likely requires efforts on both fronts.

To help guide efforts of transporting and testing EBTs, it is important to understand both how EBTs are selected and how cross-cultural adaptations are made. In the area of selection, a recent study clearly outlined a specific strategy for treatment selection in LMIC [16]. The authors specified four phases: 1) qualitative exploration of needs, 2) review of gray literature and expert consultation, 3) systematic review of the literature, and 4) consultation with local experts and stakeholders [16]. The authors highlighted that for child-focused services in LMIC, the intervention selection process is a critical but largely overlooked step.

Turning to cross-cultural adaptation of the EBT, a recent paper summarized adaptation and implementation of adult EBT in three RCTs conducted in Asia and Africa [17]. The authors noted that most core components of the adult-focused EBT (i.e., IPT and CBT) showed “universal applicability” (p. 526), with a set of relatively consistent modifications to enhance cross-cultural acceptability and delivery by non-specialist, lay counselors in a few areas. These modifications included simplifying language, reducing psychological jargon, promoting greater family involvement, using local metaphors and examples, avoiding diagnostic labels, and increasing the use of pictorial representations. With these modifications, providers found the interventions to be cross-culturally acceptable [17]. Other adult-focused papers on EBT adaptation for LMIC include CPT for use in Northern Iraq [18] and IPT for use in Southern Uganda [19]. To our knowledge, only one paper documented the adaptation of a treatment for youth within a low-resource country [20]. Verdeli and colleagues [20] presented the rationale for choosing IPT for Adolescents (IPT-A) for use in a refugee camp in Northern Uganda, discussed cultural modifications, and concluded that IPT-A was acceptable.

Psychotherapeutic interventions for children differ from adult models in some important ways that could affect the choice of an EBT and its cross-cultural adaptation. First, most child and adolescent mental health EBTs include caregivers or supportive adults working throughout the model with the youth. In LMIC, this type of person may or may not be available, and the relationship between caregiver and child may be different across cultures and contexts. Second, youth EBTs span a rapidly changing developmental time period. Working with a 7-year-old versus a 16-year-old can significantly affect how the intervention is applied. Finally, working with children and adolescents usually requires additional safety and protection steps due to the lack of independence and/or legal rights of different aged children.

There are data to suggest that child and adolescent mental health is a neglected area, with considerably fewer trials and overall implementation of programs than with adult populations [6]. Yet there have been calls for the importance of addressing child mental health in relation to the Millennium Development Goals (MDGs) and the overall reduction of the treatment gap for youth [6,21]. In order to work towards this goal and give due attention to child and adolescent mental health, more studies are needed that detail selection and adaptation specific to youth in LMIC. The goals of this paper are to 1) elaborate on the strategies used to select an EBT for symptoms of sexually abused and traumatized children and adolescents in Zambia and 2) describe the collaborative process of adapting the EBT Trauma-focused Cognitive Behavioral Therapy (TF-CBT) [22] for use in Zambia. We also present the modifications made to TF-CBT and discuss future directions. To our knowledge, this is the first description of both these processes with a child trauma EBT in sub-Saharan Africa.

### Background of the feasibility study in Zambia

Zambia has one of the highest HIV prevalence rates at 12.5% [23]. HIV/AIDS is the cause of more than half of the estimated 1.3 million orphans [24]. Orphans and Vulnerable Children (OVC) experience multiple traumatic experiences and stressors, including HIV-related stigma, abuse, poor health care, abbreviated childhoods and education, poverty, and reduced social support [25,26]. These traumatic events and stressors cause a myriad of problems including interpersonal and problem-solving skills’ deficits, mental health problems, unhealthy decision-making, and maladaptive behaviors, thoughts, and feelings [27-30].

In 2004, the Applied Mental Health Research Group (AMHR) at Johns Hopkins University [31] began their multiphase, collaborative Design, Implementation, Monitoring, and Evaluation (DIME) process in Zambia. The process is built on the principles of community-based participatory research (CBCR), an applied approach with the goal of influencing change in community health, systems, programs, and policies via collaborative researcher-community efforts at each step [32]. The steps of DIME include: 1) a qualitative assessment to identify priority problems from the local perspective; 2) the development/adaptation, translation, and validation of an assessment tool(s); 3) a population based assessment to gauge prevalence and severity; 4) the overall design of the program, including design of monitoring and evaluation; 5) the selection, adaptation, and implementation of an intervention; and 6) an assessment of intervention impact. This manuscript focuses on #5 above, although we reference other steps as background (see 31 for details on the DIME methodology).

In the DIME approach, the first step is a *qualitative study* to obtain the local population’s understanding of mental-health related problems; related causes; signs and symptoms; and what existing solutions are available. In Zambia, the qualitative study was conducted with HIV-affected children and caregivers and utilized both free listing (a convenience sample of 25 HIV-infected women and 20 HIV-affected children) and key informant interviews (N=21). Results (Step 2 in DIME) indicated a high prevalence of child trauma experiences, including sexual abuse, physical abuse, and domestic violence, resulting in trauma-related symptomatology and grief (see Table 1) [33]. A common theme across all responses was the connection between HIV and traumatic experiences and their sequelae. In the DIME methodology, this step is the foundation for all other steps and decision processes. Summarized; full results in the published study [33].

**Table 1:** Qualitative study results

**Symptoms of sexual abuse**	**Symptoms of exposure to domestic violence**	**Locally identified** “**Behavior Problems**”
Crying	Not growing up peacefully	Drinking beer
Thinking too much	Feeling lonely	Stealing
Alone and withdrawn	Imitating bad language	Having no respect
Fearful it will happen again	Looking unhappy and miserable	Being out of control
Feeling used	Looking confused	Raping others
Looking confused		Looking “scruffy”
Damaged psychologically		Not attending school
Feeling rejected		Disturbed thinking
Shy		Smoking “dagga”(marijuana)
Difficulty concentrating		
Feeling uneasy and surprised		
Having an unsettled mind – thinking about what happened		

Step 2 in the DIME process includes the development/adaptation, translation, and validation *of mental health assessment tools* for the local population [34,35]. This allows us to quantitatively confirm the presence of key symptoms in a wider population and develop valid and useful quantitative tools for subsequent screening and intervention evaluation. In Zambia, following the qualitative study, the research team adapted and tested the validity of a series of assessment tools for use with children in and around Lusaka, Zambia. These were chosen based on the results of the qualitative study and in close collaboration with local stakeholders. The assessments included: 1) the Child Post-Traumatic Stress Disorder Reaction Index Revision (PTSD-RI) [36-38], which demonstrated good reliability and validity [39]; 2) a measure on shame [40]; and 3) the Child Behavior Checklist (CBCL) [41] (manuscripts in preparation on the last two). DIME step 3 – the prevalence study – was not completed at this site.

This manuscript focuses on step 5 of the DIME methodology – the selection, adaptation, and implementation of an intervention in Zambia. Aspects of step 4 – program design, including monitoring and evaluation – will be discussed in relation to the intervention only.

## Methods

### Intervention selection

#### Meetings with local stakeholders

Following the qualitative study described above, the research team met with local stakeholders to share the results of the qualitative study and get their additional perspectives on what is currently done, as well as what should be done, to address the identified mental health problems. Stakeholders included individuals from local academic institutions, organizations working on child mental health issues, and mothers from the community. The group discussed the qualitative results and demonstrated that most local women and children did not know of services specifically for sexual abuse or domestic violence. Some mentioned that an individual could go to a “counseling center,” but they were unsure if these centers could meet non-HIV related needs. Stakeholders each presented on services they knew existed in the community, giving a description of each and the population they targeted. Local programs described included: 1) a center offering general counseling; 2) an ongoing study helping discordant HIV couples [42]; 3) HIV voluntary counseling and testing; 4) the Victims Crime Unit of the police, which officially takes reports of child abuse; and 5) a list of local non-governmental organizations (NGOs) and community-based organizations (CBOs) that offered various sport or support programs (e.g., soccer groups and drama presentations) with non-specific target populations. Stakeholders identified a void of services for moderate to severe symptoms following child abuse (e.g., sexual abuse) and death of loved ones. It was agreed that the group would reconvene after an investigation of treatment options that addressed moderate to severe symptoms of trauma and grief.

### Investigation and review of potential treatment options

Scientific literature, web-based treatment clearinghouses, and the grey literature were reviewed by the research team to identify EBTs that addressed symptoms resulting from the experience(s) of trauma and/or grief. EBTs were also reviewed for the presence of effectiveness data, including if any cross-cultural studies had been done. Potential treatments were summarized and distributed to stakeholders via handouts in country for review. Treatments that were reviewed included TF-CBT [43], KidNET ([44], Seeking Safety [45], Abuse-focused CBT [46], Parent Child Interaction Therapy (PCIT) [47], and Cognitive Behavioral Intervention for Trauma in School (CBITS) [48]. Key reports e.g. [49], articles e.g., [50], and a website [51] were also shared. Another stakeholder meeting was convened in Lusaka to discuss treatment options. Local collaborators expressed preference for: 1) a treatment that serves both children and adolescents (some interventions from the literature focused on only one age group, such as adolescents); 2) a treatment that also addressed traumatic grief, due to the high prevalence of HIV/AIDS; 3) existing cross-cultural appropriateness and/or flexibility to adapt; and 4) evidence of effectiveness. Following these preferences, the team collaboratively chose TF-CBT as the intervention to adapt, implement, and evaluate in the feasibility study.

#### *Overview of TF*-*CBT*

TF-CBT [22] is a manualized treatment designed to address the diverse problems of traumatized children, ages 4-18, and their parents or caregivers (if available). Typically, TF-CBT is brief (e.g., 12 to 16 sessions lasting 60-90 minutes each). TF-CBT includes several progressive components summarized by the acronym PRACTICE. TF-CBT components include Psychoeducation, Relaxation, Affective Modulation, Cognitive Coping, Trauma Narrative and Processing of the Trauma, In-vivo exposure (if needed), Conjoint session, and Enhancing Safety Skills.

TF-CBT has 12 RCTs supporting its efficaciousness compared to supportive therapy, treatment-as-usual, and non-directive therapy in improving children’s post-traumatic stress disorder (PTSD), depression, anxiety, shame, and behavioral problems [52]. Trials have included preschool and school-age children and adolescents [43,53,54], most of whom experienced sexual abuse and other traumatic events (i.e., poly-victimization). Longitudinal studies demonstrate maintained gains at 6, 12, and 24-months, particularly for PTSD symptoms [55-58]. Positive findings from quasi-experimental e.g., [59-61] and open trials of TF-CBT e.g, [62] further supplement the research evidence. TF-CBT has also been successfully applied cross-culturally in the United States e.g., [60,63] and in other high-income countries [64,65]. More recently, one randomized controlled trial was completed in the Democratic Republic of Congo [66] and one open trial has now been published from Zambia [67]. There are two RCTs currently running in Zambia.

### TF-CBT adaptation process

**Figure 1:**
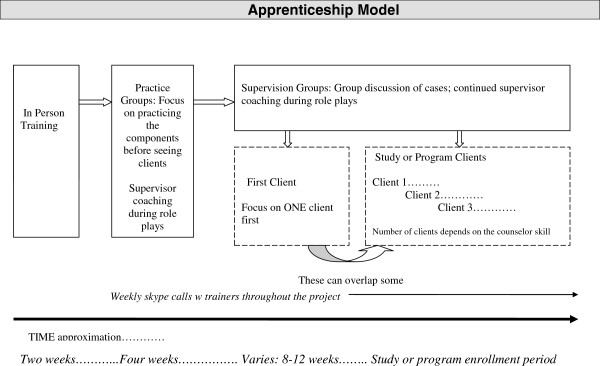
Apprenticeship model.

Treatment outcome and implementation research links fidelity of EBT to positive outcomes [68], yet there is also a need to tailor or modify EBT to be more culturally appropriate [14]. This study aimed to balance flexibility in EBT delivery (e.g., “how” components are implemented to allow for creativity in intervention strategy for individual and/or cultural needs) with fidelity to TF-CBT (ensuring delivery of core components) [69]. For adaptation and training, we used the Apprenticeship Training Model [70] (Figure 1). This training model was developed because of the lack of models for providing appropriate and ongoing training and supervision to lay mental health treatment providers, as well as clear data that “one-off” trainings are ineffective [71,72]. The essence of the model is that the treatment trainers provide ongoing supervision and training throughout the implementation process and work collaboratively with the lay counselors to adapt the model. In relation to adaptation, the role of the trainer is to pay attention to fidelity (i.e., the goals of each component), while the lay counselors and local supervisors take the lead on modifications needed to fit their culture and population (i.e., “how” the goals are accomplished and the appropriateness of goals). The local project staff, the lay counselors, and their local supervisors are thus the primary sources and experts for the EBT’s adaption, as these individuals grew up in and are part of the local community and have learned the intervention—putting them in a unique position to guide adaptation.

### Pre-training adaptation

Prior to the training of the lay counselors and their local supervisors in TF-CBT, existing training materials were adapted by the lead trainer (LM) to replace technical terms, jargon, and American idioms with simple English terms and phrases. For example, the “affective modulation” component was referred to as “talking about feelings.” Adapted materials were reviewed by a TF-CBT developer and a Zambian colleague prior to the training to ensure that simplified language did not change core component goals and that the language and concepts were understandable.

### TF-CBT training and adaptation

Two five-day TF-CBT live trainings were conducted in Lusaka, spaced by 2.5 months. All training was conducted in English, as English is widely spoken in the country. Twenty-two Zambian lay counselors (13 females; 9 males) were trained in TF-CBT. Lay counselors were students from a local university (N=7), senior officials/staff at a local university (N=2), local staff of local and international NGOs (N=11), and workers from the University Teaching Hospital and the local psychiatric hospital (N=2). Trainees had varied experience and educational backgrounds in teaching or psychology; however, only three had any formal mental health training. Nineteen of the 22 lay counselors continued after the training providing TF-CBT in addition to their existing jobs and roles; three discontinued involvement due to demands at their current positions. Three local supervisors were chosen during the training based on demonstrating advanced skill at TF-CBT and leadership abilities (e.g., others turning to them for advice and direction). One local supervisor was a lecturer at the University of Zambia, one was a counselor from a NGO, and one worked as outreach staff at a local NGO. Since these local supervisors were trained alongside the lay counselors, they all participated in the adaptation process in the same way.

The trainer (LM) didactically presented a component of TF-CBT and then modeled the component via a role-play. The Zambian trainees (inclusive of supervisors and counselors) then discussed the aspects that seemed appropriate and made recommendations for those that did not seem cross-culturally appropriate. For example, when parenting skills were discussed, all trainees felt that “time out” would likely not work due to logistics of the home (see cross-cultural section below for details). Following discussion, trainees broke into small groups (3-5 persons) to role-play the component themselves, with peer feedback. During this time, they were encouraged to try different implementation techniques they thought would work well with children in Zambia and report back to the larger group. The trainer rotated through groups to observe the role-plays, coach, and provide feedback. Following the role-plays, the larger group discussed effective and ineffective modifications. For example, while practicing “talking about feelings,” one counselor tried singing different songs for each emotion, which worked well. Another counselor tried playing a game that reportedly no one in the small group understood. The trainer’s role was to assess whether suggested modifications maintained fidelity to TF-CBT. At times, experiential training techniques were used to facilitate discussion about cross-cultural appropriateness. For example, to allow trainees to experience how receiving, or not receiving, praise feels, for one hour the trainer withheld praise or encouragement; during the next hour she provided frequent praise and encouragement. Trainees discussed these in-vivo experiences in the training.

### Post-training activities and adaptation

Following the in-person trainings, local TF-CBT practice groups were held each week for two hours led by three local supervisors (six counselors per group). Each week, counselors reviewed an agenda from the trainer with the goals of role-playing a particular component multiple times and further discussing cultural modifications. Notes were taken by the supervisor at each meeting and sent to the trainer via email, who then reviewed the modification suggestions for fidelity to the TF-CBT model. Shortly after the second training, local counselors and supervisors began participating in a one-year feasibility study on TF-CBT in which each counselor was expected to take at least one case. The primary goals of the feasibility study were to document the adaptations and assess the feasibility and acceptability of TF-CBT in Zambia. The adaptation process continued throughout the study with the local counselors and supervisors reviewing the modifications together and then presenting them to the trainer for a final “check” on fidelity to TF-CBT. The supervisors discussed modifications weekly so that these could be shared across all supervision groups.

### Fidelity monitoring and documentation of adaptation

During the feasibility study, supervisors took notes at each group meeting on TF-CBT implementation with children and caregivers, as well as any cultural modifications made. Additionally, after each TF-CBT session, counselors themselves completed case notes that required documentation of components delivered, logistics (session date, number, and duration) and details on implementation strategy (e.g., how the component was delivered, methods, activities, and adaptation). These notes were compiled and then reviewed by the training team (LM, SS) and the local supervisors (MK, MI), along with notes from the two trainings in Lusaka. A database was developed that detailed the implementation techniques across each component.

## Results

### *Cross*-*cultural modification of TF*-*CBT in Zambia*

The core components of TF-CBT were retained in Zambia. Local counselors reported in the training that they did not feel that any core components were inappropriate to the local context, although they did identify areas that needed modification. Adaptations are described in the following sections.

### Psychoeducation

TF-CBT “standard” psychoeducation goals addressed in the first session include 1) educating the family about trauma, trauma reminders, and related symptoms; 2) normalizing and/or validating traumatic reactions; 3) instilling hope for recovery; and 4) explaining TF-CBT. When children have been sexually abused, TF-CBT includes providing education about sexual abuse (e.g., prevalence, sexual body parts). In the initial training, local counselors warned that it was not customary for families to talk about anything sex-related, including naming genitals. Therefore, counselors introduced sexual education more gradually throughout the treatment and first gained permission from a caregiver. In some instances, they invited the caregiver into session to explain to the child that is was “okay and not disrespectful” to speak about sex in this particular context. While explaining the TF-CBT program, counselors also provided information about how TF-CBT differed from local “counseling.” Counselors explained that this modification was important because in Zambia the most common counseling experience is with Voluntary Counseling and Testing (VCT) for HIV. Counselors defined VCT in this context as receiving advice and usually lasting only for 1-2 sessions (5-20 minutes each).

### Parenting skills

This element required the most modification for cultural applicability. In Zambia, the parent-child relationship varies depending on the parent’s age, occupation, and cultural background (e.g., tribe). According to the counselors, Zambian parents typically use a variety of disciplinary techniques, which may include shouting, withdrawing a privilege, and/or spanking [*Zambian counselors, personal communication,* 2007]. In TF-CBT, non-physical, positive parenting techniques (e.g., effective commands, praise, and rewards) are taught. Counselors felt praise was appropriate but said it was also uncommon between caregiver and child. They collectively decided to use experiential scenarios and role-plays to introduce praise and determine caregiver perception of the utility. For instance, counselors described or role-played scenarios with mothers about a husband never giving praise for a good meal and then discussed how it felt if the husband verbalized that the meal was very good. The counselor then asked whether the parent would likely make that meal again. Counselors felt this approach was more culturally appropriate because parents may not automatically understand why one would praise a child or see the impact that praise can have on behavior. A qualitative study examining caregiver perspectives on TF-CBT indicated that praise was utilized and liked [73].

In TF-CBT, “time out,” as a non-physical disciplinary strategy, is often taught. In Zambia, time out did not seem to be appropriate and was not taught to parents, as many of the families counselors were working with lived in a one-room “hut,” and there was no place to implement time-out. Moreover, as most children cared for younger siblings, parents found it unconstructive to have older children “waste” time unproductively in time-out. Clear, positive communication and effective commands were skills that local counselors liked and felt had value, but stated a challenge that “Zambian parents do not typically communicate with their children about daily events, thoughts, and feelings” [*Zambian counselors, personal communication,* 2007]. Finally, local counselors did incorporate positive rewards, but needed to brainstorm first themselves and then with each family to identify non-tangible or non-purchased rewards for good behavior since much of the population struggled with poverty. Rewards most commonly used were: 1) release from a chore, 2) extra time with friends, 3) bringing a friend home to play, and 4) participation in extracurricular activities.

### Relaxation

This component of TF-CBT felt very appropriate to Zambians, but analogies needed to be adapted to fit the culture and local practices. Stress was equated to cooked beans that can expand and spill over the pot if the contents are not measured carefully. To teach relaxation, counselors used the analogy of “cooked” versus “uncooked” okra to conduct progressive muscle relaxation with children (versus the tin soldier/wet noodle example commonly used in the United States). Counselors also incorporated activities related to the child’s religion for relaxation such as singing gospel music, praying, and reading Bible scriptures. Other local relaxation activities included cultural music and dance, meditation, and/or cultural games identified by counselors and TF-CBT supervisors. One example of a cultural game played to relax is “Amina Kabala,” a hand game played by two people while singing a song.

### Affective modulation

TF-CBT Affective Modulation goals include understanding words that children use to describe their feelings associated with particular events and to rate the intensity of emotions across situations. Two cultural elements influenced this component. First, counselors stated that it is often uncommon for children to express feelings to parents. One Zambian author (MK) further explained the sentiments of the group: “There are a lot of cultural implications for a child to tell a parent how they are feeling—it may appear the child is ungrateful. Even if they feel bad about something they will not bring it out because they will be told that this is being disrespectful.”

Given these cultural norms around emotional expression, counselors developed a script to explain some advantages of talking openly about feelings related to sadness and trauma. Second, counselors reported that in the main languages used in Lusaka and the surrounding areas (Nyanja and Bemba), one word represents many different feelings (e.g., excitement and happiness are described by one word—Nyanja: *Kokondwela* or *Kuvela*-*Chikondi*; Bemba: *Ukumfwa Bwino* or *Ukutemwa*. To address this challenge, counselors created handouts listing terms for emotions in Nyanja and Bemba that they could discuss with children. For this component, other modifications documented included the use of traditional music to identify feelings and different sized sticks were used to scale the intensity of an emotion in different situations.

### Cognitive coping

The goals of cognitive coping include teaching that how we think about a situation impacts feelings and behavior, and that changing thoughts can positively impact feelings and behavior, even when the situation cannot be changed. Counselors noted frequent use of this skill in their own lives, both in their case notes and in supervision meetings. A cultural modification was the inclusion and reference to religion to encourage thinking in a more helpful or accurate way. For example, counselors reported using the question, “What would God want you to think?” in order to help the client come up with a more helpful thought. Most local counselors felt that this suggestion was appropriate for clients with religious beliefs and stated that this would be the majority in and around Lusaka.

### Trauma narrative (TN)

The Trauma Narrative (TN) component gradually desensitizes a child to the traumatic memory that is causing distress. Analogies are normally used to explain why talking about a traumatic event is helpful (e.g., creating a TN is like cleaning out a wound—getting all the “bad stuff” out so it can heal). Local modifications included different analogies. For example, counselors explained to the client that when you learn to ride a bicycle, it is difficult and challenging at first. You may experience emotions like fear, and you may even fall and hurt yourself, but as you practice you become more confident. The analogy of cooking beans was also used in the sense that beans are difficult to cook because the process is lengthy and “trying,” but when ready to eat, they are delicious.

To obtain traumatic details, counselors used the analogy of cooking *nshima* (a staple food in Zambia made of cornmeal). Counselors discussed the process of cooking *nshima*, which is done in stages, each of which are very important, just like each part of their story. In the training, counselors raised concerns with talking in detail about a sexual abuse incident. Counselors decided to develop scripts to explain why it is appropriate to talk about sexual body parts in this particular setting and why it is important within this component. After reading this script, parents would be asked if they would “grant permission” to the counselors to discuss the sexual abuse with the child and personally inform the child of their permission.

### Cognitive restructuring

The goal of cognitive restructuring is to teach skills to change unhelpful or inaccurate thoughts related to traumatic experiences into more helpful thoughts. Counselors used additional local analogies to dispute commonly reported thoughts. For example, to dispute “I should have known he was a bad man,” the counselor used an analogy of groundnuts (e.g., peanuts). Counselors would ask the child, “If you bought groundnuts in the shell, can you tell precisely if they are rotten inside?” or “Can you tell the shape of the nut inside a shell before you open it?” Counselors also used religion, when appropriate, asking questions such as “What would the Bible guide us to do?”

A main challenge with cognitive restructuring raised by counselors in the initial trainings, and that continued to be challenging throughout the feasibility study, was helping clients to process unhelpful thoughts related to witchcraft practices. By report of our local counselors, witchcraft beliefs came up almost exclusively as unhelpful thoughts that led to negative feelings and behaviors. These were addressed with cognitive reprocessing (rather than attempting to change a belief structure). For example, a sexually abused child may think that “I was abused because I am possessed,” or explain that “My grandfather had to abuse me to get rid of the demons because he was possessed at the time.” Local counselors first queried the families to better understand the individual’s belief system (e.g., “Tell me more about what 'possessed’ means,” or “Tell me more about how demons came to possess you”). Local counselors explained that there are many different belief systems, and this step helped them better know how to address thoughts. Questions included, “Does every possessed person sexually abuse children?” and “Are there other ways to free someone from being possessed?” Restructured thoughts included, “When my grandfather is possessed, I can go somewhere safe so that the demons tell him to do other things.” Counselors did not attempt to alter beliefs about witchcraft, but to help the child replace unhelpful witchcraft-related thoughts with more positive ones, leading to the child feeling and behaving better and feeling safer.

### Con-joint session

The con-joint session aims to share the trauma story with a supportive caregiver (if available) and increase child-guardian communication to allow the family to keep working with the skills learned in TF-CBT. Similar to the West, some children in Zambia were uncomfortable and/or afraid to read the narrative to their caregiver. Local counselors reported that this was rooted in the “newness” of open communication between parents and their children in Zambia. A modification they incorporated was the reading of a script explaining that this was a special situation when it was acceptable to ask questions and openly communicate between parent and child.

### Enhancing safety skills

For many of the children seen, there were existing and ongoing safety concerns. For example, a perpetrator may still be living in the area or the home because he is a bread-winner. For this reason, this component was often done first and reviewed each and every session. Counselors had weekly check-ins at the beginning of each session with the child and family/caregiver in order to assess the current risk and update safety plans. The safety plans included numbers for emergency contacts, locations for the nearest police post, and developing a plan with neighbors. When teaching safety skills, the counselors would often use the analogy of *kalulu* (the hare), because the rabbit is very clever and can get out of any situation. A counselor would explain that if another animal tries to trap the *kalulu*, the hare always find a way out because it is so clever. The counselor then asks the child how they can be like a *kalulu*, finding their own way out of any situation (e.g., if a perpetrator is encountered). Another modification included a significant focus on HIV. Since Zambia is known to have one of the highest rates of HIV [23], with several of the children HIV-affected, safety skills incorporated psychoeducation on HIV-related information as well as information on positive living, HIV risk behaviors, and disclosure of HIV status.

### Other modifications

#### Engagement

Engagement is critical for implementing EBTs with high-stress families [74]. Counselors felt engagement required substantial attention as this type of “talk therapy” was mostly new to Zambia. To increase engagement and decrease stigma about talk therapy, Zambian counselors often included extended family networks in their early sessions, including “aunties” (biological aunts and motherly neighbors) and grandparents, with different caregivers attending sessions. To promote ongoing engagement, counselors sent reminders and encouragement via texting, as most Zambians own mobile phones. In the early sessions, local counselors specifically acknowledged to caregivers their understanding that bringing children to therapy required not attending to necessary household tasks (i.e., selling at the market, collecting water).

### Treatment and session duration

In Zambia, session length ranged from 30 minutes to 2 hours. Local counselors explained that punctuality is not critical in the Zambian culture. Families often arrived up to two hours late for counseling, which was considered acceptable. In addition, given that some families traveled long distances for sessions, and therefore preferred fewer, longer sessions, counselors accommodated families in this manner. Consequently, counselors needed flexibility to offer both shorter sessions (when families were late) and longer sessions (for those travelling long distances). During these longer sessions, counselors often covered more than one component, thus shortening the total number of sessions. Session numbers ranged from 12 to 32 for completed cases in the feasibility study (mean=18.6, N=21). In the United States (US), therapists commonly complete TF-CBT in 12-24 sessions; thus treatment duration was consistent with US dissemination efforts.

### Feasibility study

The modified TF-CBT was piloted, and continually modified, with 21 children and adolescents during the feasibility study. Children and caregivers were referred from a center that serves youth who have experienced sexual violence—the “One-Stop Centre” [75]. Existing services at the Centre included medical exams, HIV testing, post-exposure prophylaxis, and legal interviews. Inclusion criteria for the participants was trauma exposure and a 39 or higher on the modified, locally validated measure of PTSD symptoms, the PTSD-RI [39]. The sample (N=21) was female (100%) sexually abused children with a mean age of 12.76 (SD= 1.75). Participants endorsed exposure to a number of traumatic events, in addition to sexual abuse. Children and their guardians were interviewed pre-treatment by the counselor providing treatment and post-treatment by a different counselor. Paired sample t-tests were conducted to examine the mean differences in trauma symptoms from baseline to follow-up for participants who had both a baseline and follow-up interview (N=18) (DIME process Step 6). Results revealed a significant difference in the means between baseline, (M= 72.17, SD= 37.79) and follow up (M=50.50, SD= 43.47) PTSD-RI scores, t_(17)_= 2.23, *p*<.05 (CI 1.21, 42.13).

## Discussion

This paper describes the process for choosing an EBT for children in a LMIC and the adaptation of the treatment chosen—TF-CBT. This process involves a number of steps that build on one another using community-based participatory research principles [31]. The treatment selection is a collaborative and iterative process [16]. Local community input is critical to understanding what is currently available and used in the community, as well as potential interventions that might “fit.” The “fit” of an intervention within a service system context is considered a key variable affecting implementation and acceptability [76]. TF-CBT was appealing to our national collaborators given their perception of needs, gaps in services, target age range, and effectiveness in addressing the primary problems found in the qualitative study. This collaborative selection process led to a strong buy-in from the local community, which eventually resulted in the Ministry of Health formally supporting and recommending TF-CBT in Zambia [Author JM: *personal communication,* 2010].

Like most EBTs, TF-CBT is manualized, with positive outcomes posited to be associated with high fidelity or adherence to the manual. Given the need for both fidelity and adaptation, it is critical to work collaboratively *with* local counselors and stakeholders to make culture-specific applications [77,78], rather than making these a priori, without local input. Three key lessons were learned in making effective, high-fidelity, and culturally-responsive modifications. First, the Apprenticeship Model of training [70] creates an opportunity to discuss and “pilot” cultural modifications during training, follow-up practice, and supervision groups. The emphasis on practice encourages role-plays in local languages. Counselors began exploring ways to implement TF-CBT in the local setting and “get their footing” by attempting to teach components in their own language, with the feedback of their colleagues. Counselors experimented with ways to deliver the components with flexibility, while engaging children and families. Second, in order to adapt but maintain high fidelity, local trainees need to first grasp the treatment rationale (the “why”) and the concrete goals (the “what”) of each component, before attempting to modify implementation strategy (the “how”). Third, it is important for trainers to establish a collegial/collaborative relationship with the trainees—trainers are experts on the treatment model, but trainees are experts on the local context and on how best to include local beliefs, stories, analogies, and everyday situations to adapt and effectively implement the treatment. Trainers focused on asking questions, encouraging input, and soliciting examples that would work within the local context. This finding is supported by other implementation research on the critical involvement of treatment developers, trainers, and collaboration in the implementation process [79].

The modifications made to TF-CBT in Zambia can be grouped into several major themes. First, engagement in treatment throughout included a larger family system, which is similar to other studies [17]. Gaining buy-in from the family system also included specific explanations around certain topics, such as what therapy is and how it differs from other services offered locally, as well as discussing sex. Second, local counselors incorporated story-telling or analogies into almost every component. This “fit” with the culture in Zambia, as storytelling is one of the primary methods of communication, and the analogies used were synchronized with everyday life of children in Lusaka. Additionally, the use of local languages prompted alterations, such as using one word for many emotions and using more drawings and pictures. Both of these may be particularly important with youth populations that have more limited vocabulary and more immature cognitive development than adult populations. Finally, there were some core cultural values that the local counselors brought into TF-CBT across many components, such as religious or witchcraft beliefs. Specific to child mental health interventions, one of the primary areas of debate and modification was indeed in caregiver involvement and how/when parent-child communication is appropriate. Another modification was spreading out the sexual abuse-specific psychoeducation and gaining caregiver permission to discuss this with children.

## Conclusions

This paper addresses a significant void in the literature by documenting the implementation processes of choosing and modifying an EBT, which are critical to reducing the youth mental health treatment gap in LMIC [16,80]. These processes fit within a broader Design, Implementation, Monitoring, and Evaluation (DIME) [31] methodology developed for identifying and addressing mental health problems in LMIC. Utilizing the Apprenticeship Model of training and supervision assisted in ensuring flexibility within fidelity, a topic of considerable importance in the implementation science literature [69,81,82]. This dual focus on flexibility and fidelity is increasingly being emphasized as an important aspect of provider satisfaction and adoption, as well a way to enhance the ability to provide interventions to diverse populations [83]. Future research should examine such decision-making processes and stakeholder and counselor perspectives on the intervention.

Upon review of all the case notes, the adaptations made to TF-CBT were conceptualized by the authors as “modifications in *techniques*,” rather than alterations in core TF-CBT components or goals. This is similar to other research examining adaptations of EBTs in adults [17,18,84]. More research is needed, documenting both the process of adaptation and the resulting cross-cultural modifications, particularly with children and their caregivers. In Zambia, the culturally modified TF-CBT is being tested in RCTs, allowing an opportunity to test the effectiveness of TF-CBT. In the current absence of this empirical base, EBTs should not be denied evaluation in cross-cultural contexts, particularly when there is growing evidence that supports the use and acceptability of these interventions in diverse communities abroad.

## Abbreviations

AMHR: Applied Mental Health Research Group; CBT: Cognitive Behavioral Therapy; CPT: Cognitive Processing Therapy; CETA: Common elements treatment approach; CBO: Community-based organization; CBCR: Community-based participatory research; DIME: Design, implementation, monitoring, and evaluation; EBT: Evidence-based treatment; IPT: Interpersonal Psychotherapy; IPT-A: IPT for Adolescents; LMIC: Low- and middle-income countries; mhGAP: Mental health gap action programme; MDGs: Millennium development goals; NGO: Non-Governmental Organization; OVC: Orphans and vulnerable children; PTSD: Post-traumatic stress disorder; PTSD-RI: Post-Traumatic stress disorder reaction index Revision; RCT: Randomized clinical trial; TF-CBT: Trauma-focused Cognitive Behavioral Therapy; VCT: Voluntary Counseling and Testing; WHO: World Health Organization.

## Competing interests

The authors declare that they have no competing interests.

## Authors’ contributions

LM conceived the idea for the manuscript and was the PI leading all activities. MK, and MI are local Zambians who significantly participated in the cross-cultural adaptation of TF-CBT in Zambia. LM, SD, SS, and JC discussed the fidelity of TF-CBT as it was designed. SD, PB, JB, and JC were involved in developing the research and methodology. All authors contributed to the writing of this manuscript. All authors read and approved the final manuscript.
